# Media use and brain development during adolescence

**DOI:** 10.1038/s41467-018-03126-x

**Published:** 2018-02-21

**Authors:** Eveline A. Crone, Elly A. Konijn

**Affiliations:** 1 Department of Psychology, Faculty of Social Sciences, Leiden University, Wassenaarseweg 52, 2333AK Leiden, Netherlands; 2 Department of Communication Science, Media Psychology, Vrije Universiteit Amsterdam, De Boelelaan 1105, 1081 HV Amsterdam, Netherlands

## Abstract

The current generation of adolescents grows up in a media-saturated world. However, it is unclear how media influences the maturational trajectories of brain regions involved in social interactions. Here we review the neural development in adolescence and show how neuroscience can provide a deeper understanding of developmental sensitivities related to adolescents’ media use. We argue that adolescents are highly sensitive to acceptance and rejection through social media, and that their heightened emotional sensitivity and protracted development of reflective processing and cognitive control may make them specifically reactive to emotion-arousing media. This review illustrates how neuroscience may help understand the mutual influence of media and peers on adolescents’ well-being and opinion formation.

## Abstract (web summary)

The current generation of adolescents grows up in a media-saturated world. Here, Crone and Konijn review the neural development in adolescence and show how neuroscience can provide a deeper understanding of developmental sensitivities related to adolescents’ media use.

## Introduction

Media play a tremendously important role in the lives of today’s youth, who grow up with tablets and smartphones, and do not remember a time before the internet, and are hence called ‘digital natives’^1,2^. The current generation of the adolescents lives in a media-saturated world, where media is used not only for entertainment purposes, such as listening to music or watching movies, but is also used increasingly for communicating with peers via WhatsApp, Instagram, SnapChat, Facebook, etc. Taken together, these media-related activities comprise roughly 6–9 h of an American youth’s day, excluding home- and schoolwork (https://www.commonsensemedia.org/the-common-sense-census-media-use-by-tweens-and-teens-infographic)^3,4^. Social media enable people to share information, ideas or opinions, messages, images and videos. Today, all kinds of media formats are constantly available through portable mobile devices such as smartphones and have become an integrated part of adolescents’ social life^5^.

Adolescence, which is defined as the transition period between childhood and adulthood (approximately ages 10–22 years, although age bins differ between cultures), is a developmental stage in which parental influence decreases and peers become more important^6^. Being accepted or rejected by peers is highly salient in adolescence, also there is a strong need to fit into the peer group and they are highly influenced by their peers^7^. Therefore, it is imperative that we understand how adolescents process media content and peers’ feedback provided on such platforms. Adolescents’ social lives in particular seem to occur for a large part through smartphones that are filled with friends with whom they are constantly connected (cf. “A day not wired is a day not lived”^5,8^). This is where they monitor their peer status, check peers’ feedback, rejection and acceptance messages, and encounter peers as (idealized) images^9^ on screens^5,8,10^. Likely, this plays an important role in adolescent development, and we therefore focus primarily on adolescents’ social media use^11^. Most media research to date is based on correlational and self-report data, and would be strengthened by integrating experimental paradigms and more objectively assessed behavioral, emotional, and neural consequences of experimentally induced media use.

**Fig. 1:**
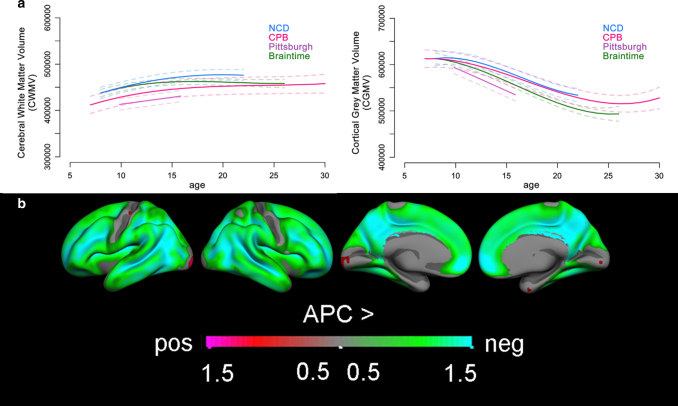
Longitudinal changes in brain structure across adolescence (ages 8–30). **a** Consistent patterns of change across four independent longitudinal samples (391 participants, 852 scans), with increases in cerebral white matter volume and decreases in cortical grey matter volume (adapted from Mills et al., 2016, NeuroImage^105^). **b** Of the two main components of cortical volume, surface area and thickness, thinning across ages 8 to 25 years is the main contributor to volume reduction across adolescence, here displayed in the Braintime sample (209 participants, 418 scans). Displayed are regional differences in annual percentage change (APC) across the whole brain, the more the color changes in the direction of green to blue, the larger the annual decrease in volume (adapted from Tamnes et al., 2017, J Neuroscience^15^)

Recently, cognitive neuroscience studies have used structural and functional magnetic resonance imaging (fMRI) to examine how the adolescent brain changes over the course of the adolescent years^6^. The results of several studies demonstrate that cognitive and socio-affective development in adolescence is accompanied by extensive changes in the structure and function of the adolescent brain^6^. Structurally, white matter connections increase, allowing for more successful communication between different areas of the brain^12^. The maturation of these connections is related to behavioral control, for example, connections between the prefrontal cortex and the subcortical striatum mediate age-related improvements in the ability to wait for a reward^13^. In addition to these changes in white matter connections, neurons in the brain grow in number between conception and childhood, with greatest synaptic density in early childhood. This increase in synaptic density co-occurs with synaptic pruning, and pruning rates increase in adolescence, resulting in a decrease in synaptic density in late childhood and adolescence^14^. Structural MRI research revealed that the peak in grey matter volume probably occurs before the age of 10 years, but dynamic non-linear changes in grey matter volume continue over the whole period of adolescence, and the timing is region-specific^15^. Interestingly, changes in grey matter volume are observed most extensively in brain regions that are important for social understanding and communication such as the medial prefrontal cortex, superior temporal cortex and temporal parietal junction^16^. Figure 1 displays the extensive changes in the human cortex during adolescence.

Given that brain regions involved in many social aspects of life are undergoing such extensive changes during adolescence, it is likely that social influences—which also occur through the use of social media as the internet connects adolescents to many people at once—are particularly potent at this age in coalescence with their media use. Also, subcortical brain regions undergo pronounced changes during adolescence^17^. There is evidence that the density of grey matter volume in the amygdala, a structure associated with emotional processing, is related to larger offline social networks^18^, as well as larger online social networks^19,20^. This suggests an important interplay between actual social experiences, both offline and online, and brain development.

This review brings together research on media use among adolescents with neural development during adolescence. We will specifically focus on the following three aspects of media exposure of interest to adolescent development^21^: (1) social acceptance or rejection, (2) peer influence on self-image and self-perception, and (3) the role of emotions in media use. Finally, we discuss new perspectives on how the interplay between media exposure and sensitive periods in brain development may make some individuals more susceptible to the consequences of media use than others.

## Being accepted or rejected online

Experiencing acceptance or rejection when communicating via digital media is an impactful social experience. Extensive research, including large meta-analyses, has demonstrated that social rejection in a computerized environment can be experienced similarly as face-to-face rejection and bullying, although the prevalence of cyberbullying is generally lower^22,23^ (and studies vary widely: prevalence rates depend on how cyberbullying is defined and measured). In all, cyberbullying peaks during adolescence^24^ and large overlap has been found between victims and bullies. In part, this overlap could be explained by victimized adolescents seeking exposure to antisocial and risk behavior media content^25^. The next subsections will describe recent discoveries in neuroscience on the neural responses to online rejection and acceptance.

### Neural responses to online social rejection

The emotional and neural effects of being socially excluded have been well captured by research involving the Cyberball Paradigm^26^ (https://cyberball.wikispaces.com/). Cyberball is a virtual ball-toss game in which the study participant tosses a ball with two simulated players (so-called confederates) via a screen. After a round of fair play, the confederates, who only throw the ball to each other, exclude the participant in the rejection condition. This results in pronounced negative effects on the participants’ feeling to belong, ostracism, sense of control, and self-esteem^26^. Even though the paradigm was not designed to study online rejection as it occurs today on social media, the findings of prior Cyberball studies may provide an important starting point for understanding the processes involved in online rejection. In fact, inspired by Cyberball, a Social Media Ostracism paradigm has recently been developed by applying a Facebook format to study the effects of online social exclusion^27^.

Using functional MRI (fMRI), researchers have observed increased activity in the orbitofrontal cortex and insula after participants experienced exclusion, possibly signaling increased arousal and negative affect^28^. In addition, stronger activity in the dorsal anterior cingulate cortex (ACC) is observed in adolescents and young adults with a history of being socially excluded^29^, maltreated^30^, or insecure attachment, whereas spending more time with friends reduced ACC response in adolescents to social exclusion^31^. This may possibly protect adolescents against the negative influence of ostracism or cyberbullying, although all these studies are correlational. Therefore, it remains to be determined whether environment influences brain development or vice versa. Moreover, ACC and insula activity have also been explained as signaling a highly significant event because the same regions are also active when participants experience inclusion^32^. Furthermore, studies with adolescents observed specific activity in the ventral striatum^33^, and in the subgenual ACC when adolescents were excluded in the online Cyberball computer game^34,35^, the latter region is often implicated in depression^36^. Thus, being rejected was associated with activity in brain regions that are also activated when experiencing salient emotions^37,38^. These studies may indicate a specific window of sensitivity to social rejection in adolescence, which may be associated with the enhanced activity of striatum and subgenual ACC in adolescence^33,36^.

**Fig. 2:**
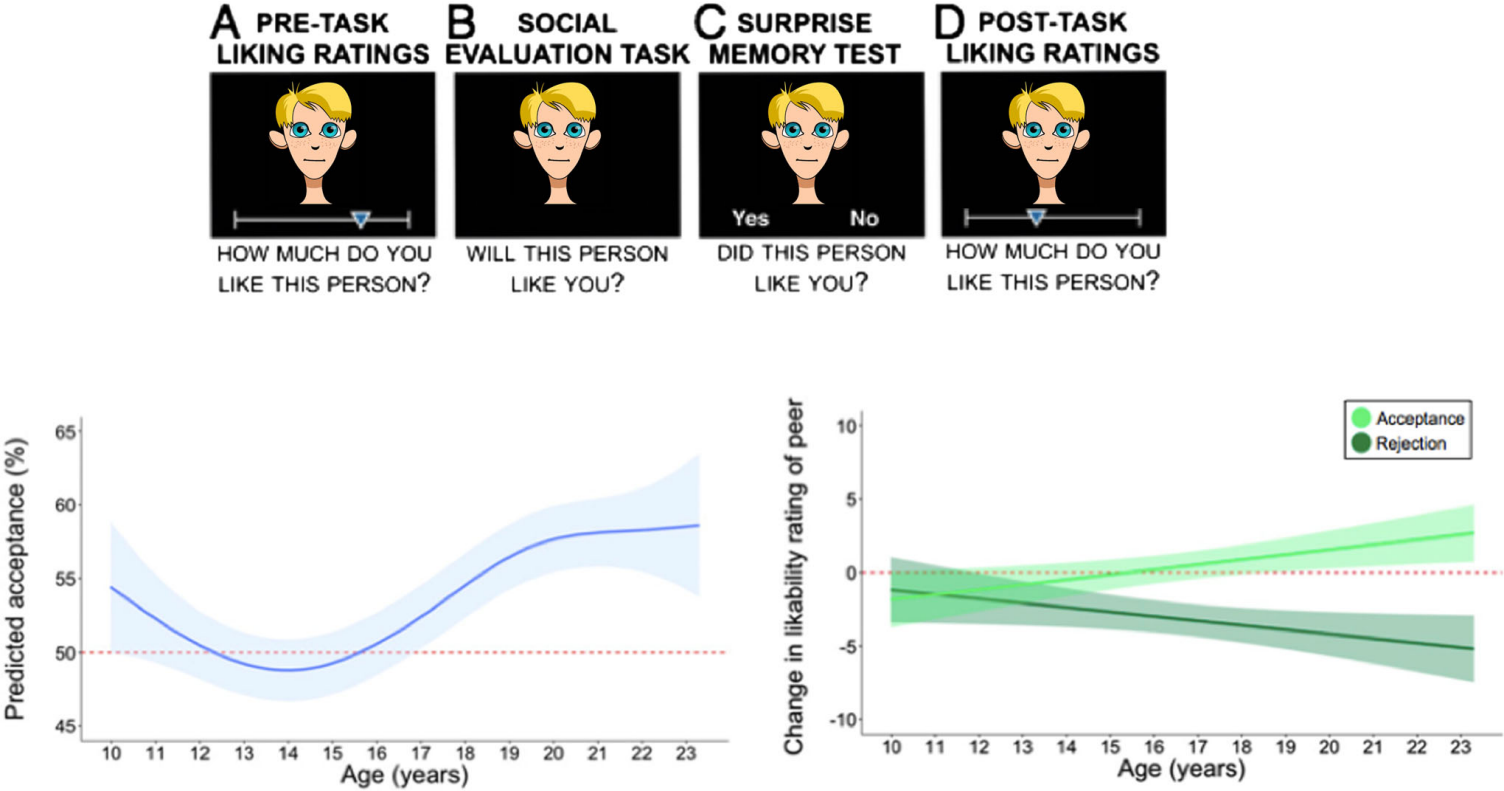
Adolescents’ expectations and adjustments of being liked and liking others. Social evaluation study in which participants between ages 10 and 23 years rated other peers on whether they liked the other person, whether they believed the other would like them, and a post scan rating of liking the other person after having received acceptance or rejection feedback from the other person. The faces used in this adaptation of figure are cartoon approximations of the original stimuli used in ref. ^40^; to see the original stimuli, please refer to ref. ^40^. The left graph shows that adolescents expect least to be liked by the other before receiving feedback (question B). The right graph shows a developmental increase in distinguishing between liking and disliking based on feedback from the other person (question D). (Adapted with permission from Rodman, 2017, PNAS^40^)

Social rejection has also been studied using task paradigms that mirror online communication more specifically. In the social judgment paradigm, participants enter a chat room, where others can judge their profile pictures based on first impression^39^. This can result in being rejected or accepted by others in a way that is directly comparable to social media environments where individuals connect based on first impression (for example,’liking’ on Instagram). A developmental behavioral study (participants between 10 and 23 years) showed that young adults expected to be accepted more than adolescents. Moreover, these adults, relative to adolescents, adjusted their evaluations of others more based on whether others accepted or rejected them, possibly indicating self-protecting biases^40^ (Fig. 2). Neuroimaging studies revealed that, being rejected based only on one’s profile pictures resulted in increased activity in the medial frontal cortex, in both adults^41^ and children^42^, and studies in adolescents showed enhanced pupil dilation, a response to greater cognitive load and emotional intensity, to rejection^43^.

Taken together, these studies suggest that adolescents show stronger rejection expectation than adults, and subgenual ACC and medial frontal cortex are critically involved when processing online exclusion or rejection. In the next section, we describe how the brain of adolescents and adults respond to receiving positive feedback and likes from others.

### Neural responses to online social acceptance

The positive feeling of social acceptance online is endorsed through the receipt of likes, one’s cool ratio (i.e., followers > following; Business Insider, 11 June 2014: http://www.businessinsider.com/instagram-cool-ratio-2014–6?international=true&r=US&IR=T.) or popularity, positive comments and hashtags, among other forms of reward^44,45^. Neuropsychological research showed that being accepted evokes activation in similar brain regions, as when receiving other rewards such as money or pleasant tastes^38^. Most pronounced activity was found in the ventral striatum, together with the ventromedial prefrontal cortex and ventral tegmental area, which is consistently reported as a key region in the brain for the subjective experience of pleasure and reward^46^, including social rewards^47^. Likewise, being socially accepted through likes in the chat room task resulted in increased activity in the ventral striatum in children^42^, adolescents^48,49^ and adults^41,50^. This response is blunted in adolescents who experience depression^36^, or who have experienced a history of maternal negative affect^51^. Apparently, prior social experiences—such as parental relations—are an important factor for understanding which adolescents are more sensitive to the impact of social media^51^. In this regard, media research showed that popularity moderates depression^10^ and that attachment styles and loneliness increases the likelihood to seek socio-affective bonding with media figures^52^.

**Fig. 3:**
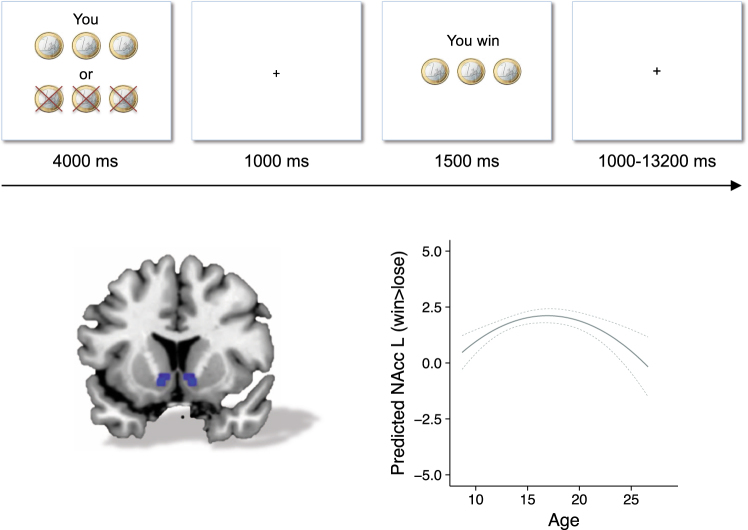
Longitudinal neural developmental pattern of reward activity in adolescence. Longitudinal two-wave neural developmental pattern of nucleus accumbens activation during winning vs. losing, based on 249, and 238 participants who were included on the first and second time point, respectively (leading to 487 included brain scans in total). A quadratic pattern of brain activity was observed in the nucleus accumbens for the contrast winning > losing money in a gambling task, with highest reward activity in mid-adolescence. (Adapted with permission from Braams et al.^55^)

Interestingly, several studies and meta-analyses using gambling and reward paradigms have reported that activity in the ventral striatum to monetary rewards peaks in mid-adolescence^53–55^ (Fig. 3; see Box 1 for views on adolescent risk taking in various contexts). These findings may suggest general reward sensitivity in adolescence such that reward centers that respond to monetary reward may also show increased sensitivity to social reward in adolescence. Social reward sensitivity may be a strong reinforcer in social media use. A prior study in adults showed that activity in the ventral striatum in response to an increase in one’s reputation, but not wealth, predicted frequency of Facebook use^56^. In a similar vein, adolescents showed sensitivity to “likes” of peers on social media^44^,^57^. In a controlled experimental study, adolescents showed more activity in the ventral striatum when viewing images with many vs. few likes, and this activation was stronger for older adolescents and college students compared to younger adolescents^57^. Thus, the same region that is active when being liked on the basis of first impression of a profile picture^48^, is also activated when viewing images that are liked by others, especially in mid-to-late adolescence, possibly extending into adulthood^57^ (see also ref. ^58^ for similar findings on music preference). These findings suggest that heightened reward sensitivity in mid-adolescence that was previously observed for monetary rewards^53^ may also be present for social rewards such as likes on Instagram. However, further research is needed to examine whether this is a specific sensitivity in early, mid or late adolescence, or perhaps this social reward sensitivity emerges in adolescence and remains in adulthood.

## Online peer influence

In addition to adolescents’ sensitivity to the feeling of belonging to the peer group^59^, the peer group also has a strong influence on opinions and decision-making^60^. Peers can exert a strong influence on adolescents through user-generated content on social media^5,61^. Co-viewing, sharing, and discussing media content with peers is common practice among adolescents in line with their developmental stage in which peers become more important than others. For example, adolescent girls often share pictures and comment on the “ideal” degree of slimness of the models they see via media when deciding how a ‘normal’ body should actually look^62,63^. Several recent neuroimaging studies, summarized below, have examined how the adolescent brain responds to peer comments about others and self, and subsequent behavioral adjustments and opinion changes. Even though not all of these designs were specific for online environments, the findings provide important starting points for understanding how adolescents are influenced by peer feedback in an online environment.

### Neural responses to online peer feedback

Neuroimaging studies in adolescents showed that peer feedback indeed influences adolescents’ behavior. Neural correlates may provide more insight in the specific parts of the feedback that drives these behavioral sensitivities^64^. One way this is demonstrated is by having individuals rate certain products such as music preference or facial attractiveness. After their initial rating, participants received feedback from others, which was either congruent or incongruent with their initial rating. Afterwards, individuals made their ratings again, and the researchers analyzed whether behavior changed in the direction of the peer feedback. Indeed, both adults and adolescents adjusted their behavior towards the group norm^58,64^, demonstrating general sensitivity to peer influence. Furthermore, when receiving peer feedback that did not match their own initial rating, participants showed enhanced activity in the ACC and insula, two regions involved in detecting norm violations^58,65^. More specifically, increased ACC activity was associated with more adjustment to fit peer feedback norms in adolescents^58^.

**Fig. 4:**
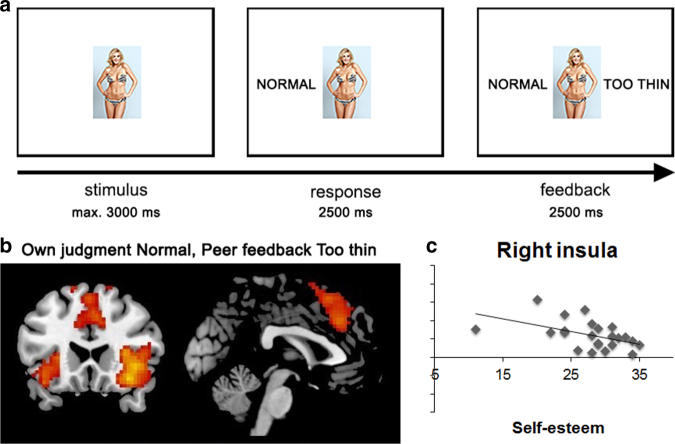
The Body Image Paradigm to study combined media and peer influence. This paradigm is designed for experiments to study the influence of peers on body image perception. **a** Participants are presented with a bikini model, and they can make a judgment whether the model is too thin or of normal weight. Their response appears on the left side of the model. Then, they are presented with ostensible peer feedback (the peer norm). **b** When this feedback deviates from their own judgment, this is associated with increased activity in dorsal anterior cingulate cortex (dACC) and bilateral insula, regions often implicated in processing norm violations. **c** Responses are larger for participants with lower self-esteem (Adapted from Van der Meulen et al.^66^)

Peer feedback effects are not only found for how individuals rate products, but also can strongly influence how they view themselves. Girls are especially sensitive to pressure for media’s thin-body ideal, and peer feedback supporting this ideal is associated with more body dissatisfaction^62,63^. We recently showed that norm-deviating feedback on ideal body images resulted in activity in the ACC-insula network in young females (18–19-years), which was stronger for females with lower self-esteem^66^ (Fig. 4). Interestingly, the girls also adjusted their ratings on what they believed was a normal or too-thin looking body in the direction of the group norm. Together, these findings suggest that peer feedback through social media can influence the way adolescents look at themselves and others.

### Neural responses to prosocial peer feedback

Interestingly, however, we also found that peer feedback can influence social behavior in a prosocial direction, for example, by having peers positively evaluate prosocial behavior that benefits the group. Neuroimaging studies of social cognition have demonstrated that thinking about other peoples’ intentions or feelings is associated with activity in a network of regions, including medial prefrontal cortex, the superior temporal sulcus and the temporal parietal junction, also referred to as the social brain network^67^. In an online peer influence study, adolescents could donate money to the group, which would benefit not only themselves but also others. Prior to the study, the participants met the other participants (confederate peers) that were not part of the group that was dividing the money. These peers, however, gave online feedback through likes on the participants’ choices. More likes were given when participants donated more to the group. This feedback was followed by higher donations^68^, and was associated with enhanced activation in the social brain network, such as the medial frontal cortex, temporal parietal junction and superior temporal sulcus^69^. Notably, the change in social brain activity in the peer feedback condition was more pronounced for younger adolescents (ages 12–13-years) compared to mid-adolescents (15–16-years)^69^. Together, these studies suggest that early adolescence may be an especially sensitive period for social media influences in risk-perception^60^ as well as prosocial directions^69^. These findings fit well with Blakemore and Mills’^6^ suggestion that, adolescence may be a sensitive period for social reorientation and social brain development, although results vary regarding whether sensitive periods are more pronounced in early or mid-adolescence. Understanding the specific sensitive windows may be important to target future interventions. Therefore, future research is needed to examine whether this is a specific sensitivity in early-to-mid-adolescence, or whether and how social reward sensitivity remains in adulthood.

## Precedence of emotions and impulsivity

A third factor that affects how adolescents process (social) media relates to the intense emotional experiences that usually accompany adolescence^70^. Emotional needs may guide adolescents’ media use and processing; for example, feeling lonely may ease the path to connect to a media figure or to rely on social media for one’s social interaction^52,71,72^. Furthermore, being engaged in media fare may evoke strong emotional reactions, such as when playing violent video games or when experiencing online rejection^73,74^. Adolescents in particular appear to be guided by their emotions in how they use and process media^5^. For example, the degree of anger and frustration experienced by early-to-mid adolescent victims of bullying was associated with increased exposure to media fare portraying antisocial, norm-crossing and risk-taking behaviors over time, making these youngsters more likely to become bullies themselves^25^. Another study showed that anger instigated a more lenient moral tolerance of antisocial media content in early adolescents but not in young adults^74^. Furthermore, adolescent victims of bullying who regulated their anger through maladaptive strategies (e.g., other-blame, rumination) showed higher levels of cyberbullying themselves^25^.

### Neural responses related to retaliation and emotion regulation

Neuroscience studies can potentially provide more insight in the moral leniency following adolescents’ anger. Neuroscience research on adolescent development has shown that the development of the prefrontal cortex, an important region for emotion regulation, matures until early adulthood^15,75^. A better understanding of the interactions between brain regions that show direct responses to emotional content, and brain regions that help to regulate these responses can possibly elucidate how adolescents regulate their behavior related to media-based interactions.

Several studies examined this question by focusing on anger following rejection. Rejected-based anger often leads to retaliatory actions. Several paradigms have also shown that adolescents are more aggressive after being rejected online. For example, they gave longer noise blasts and shared less of their resources with people who previously rejected them in an online environment^41,73,76^. More activity in dorsolateral prefrontal cortex (DLPFC) after rejection was associated with less subsequent aggression^41^ and more giving^76^, possibly indicating that increased activity in the DLPFC helps individuals to control their anger following rejection. Other research showed changes in neural coupling when young men played violent video games^77^. Thus, social rejection can evoke anger, but some adolescents may be better at regulating these emotions than others. Adolescents who regulate these emotions better show stronger activity in DLPFC, a region known to be involved in self-control^41,75^.

Applying adaptive emotion regulation strategies (e.g., putting into perspective, refocusing, reappraisal) possibly requires enhanced demands on DLPFC^78^. Possibly, the late maturation of the DLPFC, together with heightened emotional reactivity, may make adolescents more likely to be influenced by media content. For example, research showed that emotional experiences biased participants’ perception of media footage: despite being told beforehand that the footage contained fiction-based materials, they attributed significantly higher levels of realism to it under conditions of emotional arousal than in a neutral state^79^. Subsequently, participants attributed more information value to the fiction-based footage up to similar levels as to the reality-based clip.

One possible direction to better understand how adolescents deal with emotional media content is by examining parallel processes. It is likely that engaging in media is associated with multiple processes^79^ such as the fast processing of emotions associated with engagement, sensation-seeking and emotional responses to media content, as well as more reflective and relatively slower processes, such as perspective taking and emotion regulation^80^. We interpret such parallel processing as coordinated networks of an inter-related imbalance between heightened emotional responsivity and protracted development of reflective processing and cognitive control^75^. For example, adolescents show a peak in neural responsivity to emotional faces in the ventral striatum and anterior insula, compared to children and adults^81,82^. In addition, adolescents show protracted development of social brain regions implicated in perspective taking^6,83^, and flexible engagement of lateral prefrontal cortex, possibly depending on personal goals^84^. When media encounters are emotionally gripping, such parallel processing may explain why people may take (fake) information from media as real—‘it just feels real’^79^. The emotional response seems to blur the borders between fact and fake; the instantaneous response based on emotional or accompanying sensory feedback apparently takes (momentary) control precedence over cognitive reflection and biases subsequent information processing^79^. These findings may perhaps also explain how social reality can be perceived in accordance to how the world is represented in emotion-arousing, sensationalist or populist media messages, even when it concerns so-called “fake news”. In all, these suggestions call for further empirical testing, specifically also comparing adolescents and adults, in which the pattern of brain changes is combined with behavioral research and opinion formation.

Another intriguing question for future research is whether regulation or control of media-generated emotions can be trained. It was previously found that training of executive functions is associated with increased activity in DLPFC^85^, but it remains an open question whether activity in DLPFC can be influenced by (aggression) regulation training and behavioral control, and whether this results in changes in the functional and structural properties of the brain. If such training were possible, video games and immersive virtual environments might provide even more useful training environments. In this respect, promising projects are ongoing, testing the use of biofeedback videogames to help youth cope with stress and anxiety and identify physiological markers, and patterns of emotion regulation^86^. Game interventions are also developed to help children to cope effectively with anxiety-inducing situations^87^. These enrichment and training programs may also be useful to test specific media sensitivities by controlling the amount of media exposure. Such designs will have important benefits over studies examining correlations between naturally occurring behaviors and developmental outcomes, which often do not allow for control of other variables such as temperament or environmental changes.

Taken together, individuals differ in how they respond to media content, especially when these evoke emotional responses or are evaluated in an emotion-aroused state. There are only preliminary studies available that link these individual differences to brain development, but possibly the regulating role of DLPFC is important to control emotional responses to rejection, fake news, violent video games, or appealing ideals. These are all questions that need to be addressed in future research, but are highly relevant given the developmental stage and time adolescents engage with these prevalent forms of media.

## Outlook for future studies

We described research in three directions that we believe are crucial in understanding how the omnipresent use of (social) media among today’s adolescents may influence them, through the following: (1) social rejection and acceptance, (2) peer influence on opinions of self and others, and (3) emotion precedence in media use and effects. We have provided a first overview of how neuroscience research may aid in a better understanding of these influences in a mediated context. However, study results appear to vary regarding the specific adolescent age ranges; sometimes effects seem specific for early- or mid-adolescents, while in other studies adolescents and (young) adults do not differ and the indicated age ranges also vary widely (e.g., for some, ‘late adolescence’ is between 13 and 17 years old, whereas in other reports, 17–25 years of age is referred to as ‘late’, see also ref. ^88^). Most adolescent samples are relatively older, whereas early adolescents (aged 10–15) are understudied and seem of particular interest in regards of sensitivity in these three areas. Therefore, further research is needed to align specific age ranges to developmental stages.

Current media technology opens possibilities to understand sensitivities to media and peers in adolescence. For example, YouTube, Facebook, and Instagram provide excellent environments to study combined with media content and peers’ feedback in adolescence^27,89^. Moreover, such social media platforms introduced so-called user-generated content^90^ and options to present and express oneself in media environments have increased tremendously, thereby increasing media’s social functions. Taking the ethical aspects of performing social media research into account, as it can impinge on users’ privacy, social media devices also provide great opportunities to understand how media exposure affects day-to-day fluctuations in mood and self-esteem.

A critical question that remains largely unanswered is how adolescents’ abundant media use may impact them developmentally in terms of structural brain development, functional brain development, and related behavior. The scientific evidence thus far is still scarce and results are mixed^91,92^. For example, digital-screen time and mental well-being appear to be best described by quadratic functions with moderate use not intrinsically harmful^93^. Several recent studies have shown that habitual use is associated with a reduced ability to delay gratification^94^, but can also have positive consequences such as increased ability to flexibly switch between tasks^95^ and feeling socially connected^96^. Adolescents who spend more time on their mobile devices may engage less in ‘real’ offline social interactions and the consequences of these communication changes are not yet well understood. Perhaps, consequences differ among those who experience their online interactions as similar to their offline interactions, or as separate worlds. Important moderators and mediators should also be taken into account to understand how online communication is processed. Finally, being constantly online also affects sleep patterns, which impacts mood as well^97^. In all, the majority of these studies are based on self-reported new media use and outcomes. Integrating both experimental methods and neuroscientific insights may advance our understanding of who is susceptible under which circumstances to which effects, positive or negative.

## Conclusion

In this review, we described the emerging body of research focused on how new media use is processed by the still developing adolescent brain. In particular, we highlighted the neural systems that are associated with behaviors that are important for social media use, including social reward processing, emotion-based processing, regulation, and mentalizing about others^98^. As these neural systems are still underdeveloped and undergoing significant changes during adolescence, they may contribute to sensitivity to online rejection, acceptance, peer influence, and emotion-loaded interactions in media-environments. In future research, it will be important to understand these processes better, especially the specific developmental sensitivities, as well as to understand which adolescents are more and less susceptible for beneficial or undesirable media influences.

The review of the literature suggests that peer sensitivities are possibly larger in adolescents than in older age groups. Peer influence effects have been well demonstrated in adolescent decision-making research, showing that adolescents take more risks in the presence of peers and when peers stimulate risk-taking^99^. This seems to hold similarly for peer influence online through online comments, also with less risky behaviors^62^. These findings have been interpreted to suggest that adolescents have a strong need to follow norms of their peer group and show in-group adherence^100^. There is a strong need for studies that experimentally test whether increased influence of peers, possibly through developing social brain regions, combined with strong sensitivity to acceptance and rejection, makes adolescence a tipping point in development for how social media can influence their self-concept and expectations of self and others. It is likely that these sensitivities are not related to one process specifically, but the combination of developmental brain networks and associated behaviors^75,84^. A critical question for future research is how neural correlates observed in this review predict future behavior or emotional responses in adolescents.

Social media have at least the following two important functions: (i) socially connect with others (the need to belong) and (ii) manage the impression individuals make on others (reputation building, impression management, and online self-presentation)^98^. The emerging trajectory of acceptance sensitivity, peer ‘obedience’, and emotion precedence may make adolescents specifically susceptible to sensationalist and fake news, unrealistic self-expectations, or regulating emotions through adverse use of media. Important questions for future research relate to unraveling whether adolescents are more sensitive to these news items than children and adults, who is most sensitive to which kind of media influence, how (one-sided) media use may influence adolescent development over time, and understand not only the risks but also how media provides opportunities for positive development, such as engaging with friends, forming new peer relations, and experiment with uncertainties or overcoming fears. Studying the interplay between media use and sensitive periods in brain development will provide important directions for understanding how media may impact youth and who is most vulnerable and under which conditions. Key questions for future research are to understand whether recent changes in media usage, delivery, dosage, and levels of engagement (e.g., as more active creators and participants, for example) are leading to different or amplified neural responses in adolescents relative to adults. Using longitudinal research, it will be important to test whether there is evidence that the still developing adolescent brain is more sensitive to, or more likely to be shaped by these changing patterns of media usage.1

### Box 1 Multiple perspectives on adolescent risk-taking

Adolescence is often defined as a period of increased risk taking and sensation-seeking, this is observed across cultures^101^ and across species^102^. However, the way risk-taking is expressed differs across generations. In middle ages, risk-taking in adolescence took place through reckless fights and wars. In contrast, in the late 20th century and early 21st century, adolescents were more prone towards risk-taking in context of alcohol, sex, and drug experimentation^103^. Recently, through social media, new forms of risk-taking are expressed, such as excessive or unlimited self-disclosure or sexting^104^. These observations suggest that social media may be the new way in which sensation-seeking behavior is expressed, which is possibly an adolescent-specific tendency to explore and learn to adapt to new social environments.
